# Incorporating Lymphovenous Anastomosis in Clinically Node-Positive Women Receiving Neoadjuvant Chemotherapy: A Shared Decision-Making Model and Nuanced Approached to the Axilla

**DOI:** 10.3390/curroncol30040306

**Published:** 2023-04-03

**Authors:** Daniel Ben Lustig, Claire Temple-Oberle, Antoine Bouchard-Fortier, May Lynn Quan

**Affiliations:** 1 Department of Surgery, Foothills Medical Centre, Calgary, AB T2N 2T9, Canada; 2 University of Calgary, Calgary, AB T2N 1N4, Canada

**Keywords:** lymphovenous anastomosis, lymphedema, breast cancer, neoadjuvant chemotherapy, decision making, axillary lymph node dissection

## Abstract

Introduction: Lymphedema remains a risk for 13–34% of breast cancer patients who require an axillary dissection (ALND) and radiation. Immediate lymphovenous anastomosis (LVA) may mitigate lymphedema by up to 30% by restoring the physiologic lymphatic drainage immediately after ALND. Currently, completion of ALND (cALND) versus radiation after neoadjuvant therapy (NAC) is being addressed by the Alliance A11202 trial, leaving a paucity of data to guide practice. Our study describes the implementation process of LVA into clinical practice after NAC for node-positive breast cancer in the current clinical context. Methods: We reviewed a prospective database of LVA in node-positive patients (cT1-4,Nany) who received NAC followed by axillary surgery ± immediate LVA from October 2021 to 2022. The evolution of the surgical approach is described. Specifically, patients who downstaged to clinically negative nodes post-NAC were offered targeted SLNB with dual-tracer and intraoperative frozen section (FS). Patients were reminded that the standard of care for any node positive is cALND. Immediate cALND with LVA was performed for grossly positive nodes or all positive SLNs; cALND was omitted for those with negative SLNs. For a microscopic disease on a frozen section, a shared decision was made pre-operatively, given each patient’s differing valuations of the benefit and risks of cALND ± LVA versus no cALND with planned regional radiation postoperatively. LVA was offered as an option as part of our institutional evaluation of the procedure. Results: A total of 15 patients were included; the mean age was 49.9 (range 32–75) with stage IIA to IIIB breast cancer. Of these, 6 (40%) were triple negative, 5 (33.3%) HER-2 positive, and 4 (26.7%) ER/PR+ HER-2 negative. There were 13 women (86.7%) who had persistent axillary adenopathy based on clinical and/or ultrasound assessment, with 8 patients proceeding directly to ALND with LVA. Among these patients, 3 (37.5%) had pathologic nodal disease, and 5 (62.5%) were node negative, confirming the limitations of pre-operative imaging. As a result, the subsequent 7 (46.7%) underwent targeted SLNB with FS, with 3 patients (42.9%) avoiding an ALND as a result of a negative FS. A total of 4 patients (57.1%) had 1 or more positive lymph nodes on FS: 3 proceeded with a cALND and LVA, and 1 patient (14.2%) opted for no cALND based on a pre-operative discussion and received adjuvant radiation and chemotherapy. Of the 11 patients who underwent ALND and LVA, 1 patient (9.1%) developed lymphedema at 6.9 months following their surgery. The accuracy, sensitivity, and specificity of pre-operative US were 46.7%, 85.7%, and 12.5% and intraoperative FS were 88.0%, 72.7%, and 100%, respectively. Conclusions: As adjuvant nodal radiation and systemic therapy continue to improve, the benefit of a cALND in patients with the limited residual disease remains unclear as we await the outcomes from clinical trials. In the era of clinical uncertainty, we propose a nuanced approach to the axilla by utilizing a shared decision model with patients, incorporating targeted SLNB with FS and completion node dissection when required and desired by the patient, coupled with LVA in a simple stepwise treatment pathway.

## 1. Introduction

Breast cancer-related lymphedema (BCRL) remains a significant morbidity in approximately 13–34% of women who require an axillary lymph node dissection (ALND) and/or adjuvant radiation as part of their cancer treatment [1,2]. Breast cancer survivors who develop lymphedema have a measurable reduction in their quality of life and are more prone to developing infections, making efforts to reduce BCRL paramount [3]. Prophylactic lymphovenous anastomosis (LVA) is a strategy that aims to mitigate the development of BCRL by restoring the physiological lymphatic drainage of the upper extremity at the time of axillary dissection [4]. This strategy has also been described in locoregionally advanced melanoma both in the axilla and the groin [5]. Studies have shown that prophylactic treatment with LVA can reduce BCLR by 30%, leading to a significant improvement in objective and patient-reported outcomes [6]. However, incorporation of this novel technique into patient care pathways has been challenging, particularly in women with positive axillary lymph nodes diagnosed pre-operatively. Women with clinically positive axillary lymph nodes detected prior to definitive treatment may be offered upfront breast surgery with concurrent ALND. Alternatively, pre-operative neoadjuvant chemotherapy (NAC) can be used in an attempt to downstage the axilla and perform a sentinel lymph node biopsy (SLNB) to determine if the metastatic disease remains in the axilla, thus avoiding the morbidity of an ALND [7,8,9]. Patients with the residual nodal disease are thought to represent chemo- and radioresistant cancer. Consequently, current guidelines recommend nodal clearance by ALND (cALND) for women with persistently positive nodes following NAC [10]. However, as the effectiveness of adjuvant nodal radiation and systemic therapy continue to improve local-regional control and overall survival benefit, there remains clinical equipoise on the need for the completion of ALND in patients with limited residual disease. Currently, the Alliance A11202 trial (NCT01901094) is accruing patients to determine whether nodal radiation alone is inferior to ALND and nodal radiation in breast cancer patients (cT1-3 N1) who have positive sentinel lymph nodes following NAC [11]. In the absence of clear evidence, we propose a nuanced approach for managing the axilla that incorporates current evidence, patient preference, and the introduction of prophylactic LVA to reduce the risk of BCRL. Herein, we describe our experience and the development of a shared decision model for patients with clinically node-positive breast cancer who received NAC that incorporates the option of LVA in a simple stepwise treatment pathway.

## 2. Materials and Methods

### 2.1. Study Participants

A prospectively collected LVA database at our institution was queried for all patients 18 years or older with clinically node-positive breast cancer who underwent neoadjuvant chemotherapy followed by definitive breast (mastectomy or breast-conserving surgery) and axillary surgery between October 2021 to October 2022. Patients were excluded from this study if they previously had ipsilateral breast and/or axillary radiation or surgery. This work was approved in accordance with our institution’s research ethics board. 

### 2.2. Patient Discussion Prior to Definitive Surgery

Following the completion of NAC, patients were re-staged by clinical examination as well as breast and axillary ultrasound by dedicated breast radiologists. Patients were categorised as either (1) clinically and sonographically node negative or (2) persistently abnormal nodes on ultrasound with or without clinically positive nodes. Patients were reminded that the standard of care for any node-positive disease following NAC was an ALND, and the option of LVA at the time of surgery was offered if the patient required an ALND. A pre-operative discussion was conducted with all patients and the breast surgical oncologist to discuss the options for axillary management. Specifically, patients whose axilla clinically downstaged to node negative post-NAC were offered a “targeted SLNB” where the previously biopsied positive node was removed with seed localization and an SLNB with dual-tracer (patent blue dye and Technicium-99 radioisotope) was performed with a minimum of 3 nodes removed. Recognising the known pCR rates by receptor profile, a proportion of women that are classified as downstaged clinically post NAC will persist with the axillary disease, all SLNs were evaluated with intraoperative frozen section (FS). In order to provide a patient-focused approach to a single definitive operative procedure, a pre-op shared decision-making discussion was undertaken to direct the next steps pending the results of the FS. In general, each outcome was reviewed; patients with no residual axillary disease on SLNB had no further surgery. Patients with all sentinel lymph nodes positive for residual macrometastatic disease on targeted sentinel lymph node biopsy were recommended a cALND with the option of LVA. For patients with a low burden of disease and thus a lower likelihood of additional positive nodes, such as those with micro- or macrometastases in only a proportion of nodes (i.e., 1 to 2 SLNs on FS), a shared decision was made pre-operatively given each patients’ differing valuation of the benefit and risks of cALND ± LVA, versus no cALND. Specifically, a fulsome discussion reminded patients that the standard of care for any residual disease would be to perform a cALND. Our discussion also acknowledged the equipoise regarding the benefit of cALND + regional radiation therapy given the current lack of evidence to date that demonstrates an improved survival which is pending the results of the Alliance A11202 clinical trial. The natural history and management of lymphedema was discussed along with the use of LVA to mitigate BCRL. Furthermore, the option of intraoperative FS for SLNB versus awaiting final pathology in which the later would result in a second procedure and a delay in radiation if necessary was reviewed. Patients with inflammatory breast cancer were only offered ALND with the option of LVA.

### 2.3. Lymphatic Reconstruction

Each patient underwent dual lymphatic mapping using indocyanine green (ICG) to the ipsilateral thenar webspace and Patent Blue Dye (2 to 4 mL) to the inner upper arm up to 15 min before the LVA. We inject with 1 mL and add additional 0.5 mL of aliquots as needed. The volume used ranges between 2 to 4 mL. Direct visualization and a SPY were used to map afferent lymphatics in the axillary basin. The plastic surgeon(s) were present throughout the operation to protect length of the lymphatics laterally during the dissection and to encourage protection of veins for anastomoses. Once the packet of nodes is removed, a decision is made for end-to-end or end-to-side anastomosis. A description of the LVA is described previously [4]. In general, end-to-end coaptation of lymphatics in an intussusception fashion is carried out under the operative microscope using a 10-0 nylon. Multiple lymphatics are coapted to an anterior branch of the axillary vein or the thoracodorsal vein. Patency is affirmed by visualizing blue dye egress as well as indocyanine green (ICG) migration into the vein.

### 2.4. Evaluation for Lymphedema

Patients were evaluated for lymphedema at regular 6 month follow up visits where they would complete a patient-reported outcome measure (LYMQOL-arm) [12] to assess their upper extremity function. They are reviewed for cording and shoulder range of motion. Their arms were measured from wrist to axilla every 4 cm, and a truncated cone calculator is used to assess volume [13]. If the limb volume difference exceeds 10% in the operative limb, the patient is referred to the lymphedema clinic for physiotherapy and compression.

## 3. Results

### 3.1. Overview of Patient Demographics, Breast Cancer, and Initial Evaluation

During the study period, 20 patients with breast cancer presenting with the clinically positive nodal disease who had LVA discussed as part of their treatment plan were identified. Within this cohort, 15 patients received NAC and were included in our study. A total of 5 patients were excluded: 4 patients did not undergo NAC, and 1 patient had been previously treated for ipsilateral breast cancer and presented with a nodal recurrence. The mean age was 49.9 (32–75), with the majority of patients presenting with stage II, triple-negative invasive ductal carcinoma (Table 1). At presentation, 13 patients had clinically palpable axillary lymph nodes (81.3%) of which 4 (30.8%) had matted lymph nodes, and 9 patients (69.2%) had mobile axillary nodal disease. All 15 patients had abnormal-appearing axillary lymph nodes in US, which were biopsy proven to contain metastatic disease prior to the initiation of NAC.

### 3.2. Reassessment Post-Neoadjuvant Chemotherapy and Definitive Surgery

**Figure 1:**
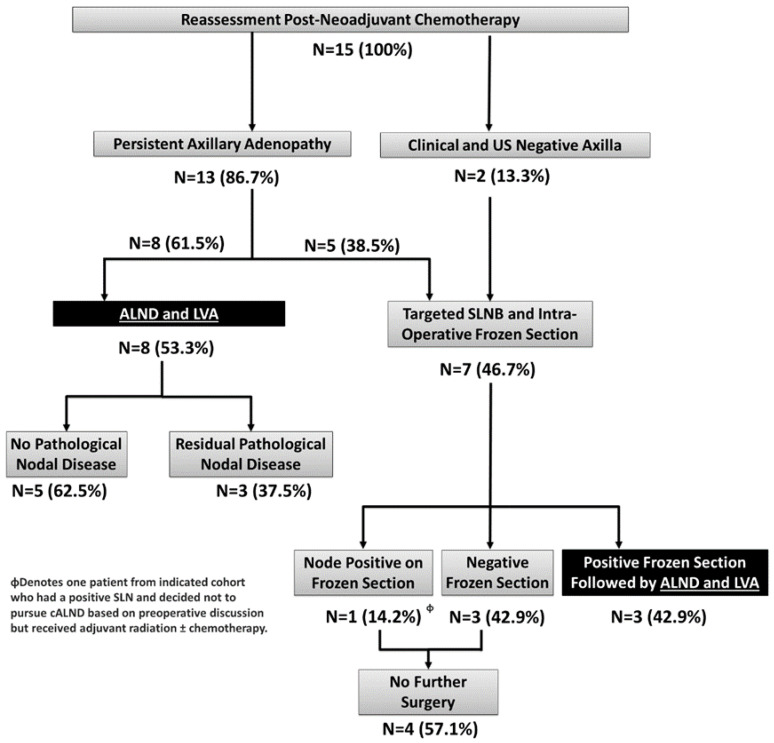
Treatment Overview and Pathological Outcomes Following Axillary Surgery. ALND: Axillary Lymph Node Dissection. LVA: Lymphovenous Anastomosis. SLNB: Sentinel Lymph Node Biopsy. US: Ultrasound.

Patients were re-evaluated by a physical exam and a repeat ultrasound of the breast and axilla following the completion of their systemic treatment. A total of 2 patients were found to have clinically and radiologically negative axillae (13.3%), while the remaining 13 (86.7%) had sonographic evidence suggestive of residual axillary disease with or without palpable adenopathy. (Figure 1). A total of 5 patients underwent a mastectomy (33.3%), 9 underwent breast-conserving surgery (60%), and 1 had axillary surgery only (6.7%) due to an occult breast primary presenting exclusively with nodal disease. With respect to axillary surgery, 53.3% of our patients (*n* = 8) had an upfront ALND followed by immediate LVA, and 46.7% (*n* = 7) had a targeted SLNB with an intraoperative frozen section. Among the patients who had a targeted SLNB with an intraoperative frozen section, 3 (42.8%), based on our pre-operative discussion, had a cALND with immediate LVA for any residual positive disease found on the frozen section. Additionally, 3 patients had a targeted SLNB and no further surgery due to a negative FS (42.8%), and 1 (14.4%) patient had a positive FS with a low burden of disease (1 of 3 SLNs with a 3 mm focus of disease) and had declined to have a cALND based on our pre-operative discussion unless all nodes were positive (Figure 1 and Table 1, Supplementary Data). The mean and range of the number of nodes removed were 13.8 and 4–28, respectively.

The median number of lymphovascular anastomoses for patients who underwent an ALND was 2 (range 1–4), with the majority using an intussusception end-to-end technique. All patients received adjuvant radiation treatment to the regional lymph node basin with or without further adjuvant systemic therapy at the discretion of the medical oncologist. 

### 3.3. Assessment of Pre-Operative US and Intraoperative Frozen Section for Predicting Positive Sentinel Lymph Nodes

Among the 13 patients who had residual axillary disease reported on the repeat US following NAC only, 46.2% (*n* = 6) had nodal metastasis detected on the final pathology revealing the accuracy, sensitivity, and specificity of pre-operative post-NAC US to be 46.7% (95% CI 21.3–73.4%), 85.7% (95% CI 42.1–99.64%), and 12.5% (95% CI 0.32–52.7%), respectively. Furthermore, for the 7 patients who had a targeted SLNB with an intraoperative SLNB frozen section, the accuracy, sensitivity, and specificity of this evaluation was 88.0% (95% CI 68.78–97.45%), 72.7% (95% CI 39.03–93.98%), and 100% (95% CI 76.8–100%), respectively. 

### 3.4. Clinical Outcomes

Patients were followed for a median of 20.0 months (15.8–27.0), and there have been no locoregional recurrences. A patient with triple-negative breast cancer who had clinically downstaged from cT2N1 to ypT1cN0 developed brain metastasis 18.4 months after surgery. None of the 4 patients who underwent a targeted SLNB alone developed lymphedema. Among the 11 patients who underwent a cALND followed by immediate LVA, 1 patient (9.1%) developed pitting lymphedema from axilla to hand, which occurred 6.9 months after the initial operation. Their limb volume discrepancy was measured at 15.5%. They were referred to the lymphedema clinic, where they underwent decongestive physiotherapy and had a compression sleeve and gauntlet fashioned. Their resultant limb volumes differential stabilised at 160 mL (7%). No infectious or other complications have arisen from their lymphedema.

### 3.5. Patient-Reported Outcomes

Patient-reported outcomes based on the LYMQOL-ARM survey filled out post-operatively between 6 to 12 months are shown in Table 2. The overall quality of life was rated high among all patients (8.1/10 ± 0.9), and in terms of function, appearance, symptoms, and mood domains, all patients had low scores, indicating minimal to no symptoms of lymphedema following axillary surgery. The single patient who developed lymphedema had a slightly higher symptom score compared to the rest of the patients who did not develop lymphedema (2.3/4 vs. 1.4/4).

## 4. Discussion

Current guidelines recommend women with clinically positive axillary lymph nodes, who have residual disease following NAC, undergo an ALND followed by regional nodal radiation [10]. Patients who undergo ALND and radiation have a 30% risk of developing BCRL, which carries significant life-long morbidity [1]. Currently, there is a paucity of clinical data to determine whether residual positive sentinel lymph nodes following NAC require axillary clearance with a formal node dissection or whether nodal radiation is sufficient to treat any remaining disease. The Alliance A11202 trial is currently accruing patients to address this question to guide management in patients with a low burden of residual axillary disease (NCT01901094, [11]). Our centre has begun to offer women prophylactic LVA at the time of ALND to help mitigate the development of BCRL. Since we began offering this technique, our approach to treating women with clinically positive nodes who received NAC has evolved to incorporate targeted SLNB with intraoperative FS, as we found that 86.6% of the patients (*n* = 13) had clinically palpable and/or suspicious nodes on the US after chemotherapy, but >50% had a complete pathological response on final pathology. For example, the initial 7 patients in our study all had abnormal-appearing lymph nodes on the axillary US and 5 of them had palpable lymph nodes following the completion of their chemotherapy. Based on their pre-operative findings, they were all offered an ALND and immediate LVA. A review of the final pathology found that 4 patients had a complete pathological response (57.1%), only 1 patient (14.3%) had the macroscopic disease (1 of 16 lymph nodes), and of the remaining patients, 1 patient (14.3%) had residual isolated tumour cells (ITCs) in 2/10 nodes, and 1 patient (14.3%) had the microscopic disease (1 micrometastasis and 2 nodes with ITCs of 28 lymph nodes). An overview of the nodal pathology for patients is demonstrated in Supplementary Data Table S1. These early results highlighted the limitations of re-evaluating the axilla with sonography and basing the decision to offer upfront ALND as opposed to a targeted SLNB on imaging alone. 

**Figure 2:**
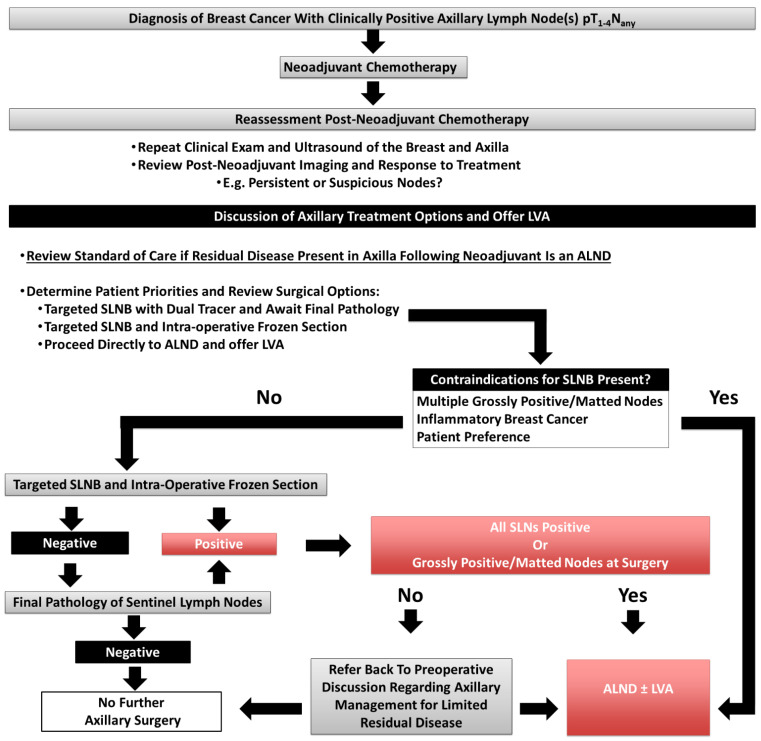
Proposed Patient Care Pathway for Patients with Clinically Positive Nodal Disease Who Received Neoadjuvant Chemotherapy. ALND: Axillary Lymph Node Dissection. LVA: Lymphovenous Anastomosis. SLNB: Sentinel Lymph Node Biopsy.

In our study, the accuracy of the pre-operative axillary US to correctly categorise the status of the lymph nodes was poor at 46.7%, which is comparable to 51.9% reported in the ACOSOG Z1071 trial [9]. However, at our institution, we had a lower specificity (12.5%) and a higher sensitivity (85.7%) compared to the Z1071 trial, which was 34.6% and 78.8%, respectively. Consequently, the 4 patients who had a complete pathological response would have avoided an ALND if we had proceeded first with a targeted SLNB and FS. From these early results, we elected to develop a personalised approach to the axilla that would take into account the treatment response to NAC based on the clinical exam and repeat imaging, as well as the patient’s preferences after a detailed discussion of the available surgical options and their risk and benefits (Figure 2). Specifically, the surgical options include proceeding directly to ALND with or without LVA, performing a targeted SLNB with dual tracer and awaiting final pathology, or performing a targeted SLNB with dual tracer and an intra-operative frozen section and deciding on cALND based on the nodal status and our pre-operative discussion with the patient. Importantly, cALND was omitted for patients with a negative SLNB on FS. For patients with the microscopic disease identified on FS, a shared decision was made pre-operatively after exploring the patient’s valuation of the benefit and risks of cALND ± LVA versus no cALND given the paucity of clinical data pending the results of the Alliance A11202 trial. Patients were reminded that the current standard of care for any node-positive disease following NAC was an ALND, and the option of prophylactic LVA at the time of surgery was offered. Patients with the residual disease were treated with nodal radiation with or without adjuvant systemic therapy at the discretion of the medical oncologists. 

After implementing the shared decision model utilizing the option of a targeted SLNB with intraoperative FS in the remaining 8 patients of our study, 3 patients (37.5%) with abnormal appearing nodes in the US had negative FS and, on final pathology, had a complete pathological response thus avoiding a cALND. A total of 4 patients (50%) had 1 or more positive nodes on FS and, based on pre-op discussion, 3 underwent cALND with immediate LVA; 1 patient (12.5%) elected to forego the cALND based on our pre-operative discussion in which they elected to avoid a dissection with a low burden of disease (1 macrometastasis in 1/3 nodes on FS) with the final pathology confirming the macrometastasis and identifying 2 additional nodes with ITCs. Lastly, 1 patient (12.5%) with inflammatory breast cancer had proceeded directly to a modified radical mastectomy with prophylactic LVA in which they had 0 of 10 lymph nodes despite multiple abnormal and enlarged nodes seen on the pre-operative US. Thus, incorporating targeted SLNB with intraoperative FS offers the benefit of knowing the nodal status in real-time, allowing for decisions to be made at the index surgery based on pre-operative discussions with the patient and providing a single definitive surgery. Less ideally, if the final pathology identifies additional positive nodes (false negative FS), a cALND with or without LVA can be offered through a second procedure to clear the axilla which delays radiation and can make the LVA more challenging. 

At our institution, intraoperative FS had a high accuracy and specificity (88% and 100%) and an acceptable sensitivity of 72.7% for all nodal diseases (ITCs and micro- and macrometastasis), which is comparable to other studies [14]. The three false negatives on FS were determined to be ITCs on final pathology, which are a known limitation for FS analysis [15]. Lastly, given the low accuracy of US following NAC proceeding initially with a targeted SLNB with dual tracer and intraoperative FS offers the benefit of preventing an unnecessary ALND in patients with an abnormal US, who, in fact, have no axillary disease. Furthermore, in patients with a normal sonogram, an intraoperative FS may identify the disease, allowing for definitive axillary clearance and immediate LVA, thus preventing the need for a second surgery and delaying adjuvant treatment while waiting for the final pathology to determine the nodal status. Our preference is to proceed with the SLNB first and will follow with the breast portion of the surgery while the FS is being analysed. Thus, our approach does not add additional time to the overall procedure. 

In our study, 11 of 15 patients underwent an ALND with immediate LVA followed by adjuvant nodal radiation. Moreover, 1 patient (9.1%) had developed lymphedema within the median follow-up period of 20.0 months, which is lower than the 20–30% reported in contemporary studies [1,16]. However, our study was not specifically designed to compare ALND with and without LVA directly. Furthermore, our median follow up was only 20.0 months; thus, it is conceivable that more patients may develop lymphedema in the future and longer follow up is needed. However, the majority of patients will develop lymphedema within the first 12–24 months, and all of our participants fell within this follow-up range [1,17]. 

Our proposed approach to the axilla has its limitations and special considerations when incorporating it into clinical practice. First, it requires collaboration with an experienced plastic surgeon trained in microvascular reconstruction and LVA. Furthermore, it requires the plastic surgeon to be available immediately following the ALND during the index case, which, if performed, adds approximately 60 min to the procedure. Unfortunately, there will inevitably be instances where the reconstruction team will be on standby, and no ALND/LVA is required based on a negative SLNB FS. In our centre, we employ a “swing room” model, which allows maximal efficiency of the plastic surgeon where they will join the case for the LVA portion of the procedure if needed and are often operating in another theatre when the surgical oncology team is performing the initial breast and axillary surgery. Moreover, the cost of additional operative time needed for the LVA is small in comparison to patient morbidity as well as the society cost in our publicly funded system to treat permanent lymphedema. A cost–benefit analysis by Johnson et al. (2021) supports that immediate LVA is a cost-effective approach to mitigate BCRL [18]. Similarly, pathologists must be available at the time of surgery to prepare and review the specimens from the intraoperative FS, which may be a challenge at some institutions. When considering our approach to the axilla, it is imperative that the patient is fully informed that the current guidelines recommend a cALND for any residual disease in the axilla following NAC. Furthermore, the potential limitations of the physical exam, US, and intraoperative FS to accurately predict residual disease following NAC should be discussed with the patient. This will allow the surgeon and patient to make an informed decision regarding how best to interpret and incorporate this information when deciding how to manage the axilla (whether to proceed directly to ALND or offer a targeted SLNB with or without an intraoperative FS). This requires the surgeon to have an understanding of how accurate the pre-operative US and intraoperative FS are at identifying positive lymph nodes at their respective institutions. Lastly, patients should be made aware that the patent blue dye used for mapping the lymphatics for LVA leaves a permanent mark on their upper inner arm. 

In summary, our approach to axillary management of breast cancer patients who received NAC for the clinically node-positive disease has several advantages. First, it may prevent unnecessary ALNDs in patients with clinically and/or sonographically positive axillary lymph nodes post-NAC by confirming SLNB positivity at the index surgery as opposed to relying solely on the pre-operative studies. In our cohort, 62.5% of patients who underwent an ALND for the suspected residual axillary disease had a complete pathological response and would have avoided this if they had a targeted SLNB. Secondly, knowing the status of the sentinel lymph nodes at the index operation, using intraoperative FS allows for definitive surgery with ALND ± LVA if indicated. This approach prevents the need for a second surgery which ultimately delays adjuvant radiation while waiting for the final pathology to determine if there is residual nodal disease and an additional procedure is required. In this present study, 3 of 7 patients who had targeted SLNB (42.9%) had 1 or more positive SLNs and immediately had an ALND and LVA at the index surgery. With respect to LVA, performing the anastomosis at the index surgery makes it easier to identify and preserve critical lymphatics in a virgin operative field as opposed to returning to the axilla in a second procedure where scar tissue has begun to form and lymphatics have been disrupted, making it more challenging to find suitable vessels and lymphatics for re-anastomosis. Thus, performing the LVA during the initial operation increases the likelihood of success for re-anastomosis and ultimately maximises the chance of preventing lymphedema. Lastly, have a pre-operative discussion regarding the clinical equipoise of axillary management of patients who undergo NAC with limited residual nodal disease empowers the patient to make a more informed decision by exploring their valuation of the benefits and risks of a cALND in this setting. Our pre-operative discussion informs the patient that the current standard of care recommends a cALND for any residual disease, but given the lack of RCT data that demonstrates omitting ALND in lieu of axillary radiation leads to worse outcomes, we feel it warrants a personalised decision for the patient. Lastly, incorporating the option of LVA for women may help them avoid decision regret should they choose surgical clearance of all axillary nodes, as their choice has been coupled with the knowledge that they added a lymphatic reconstruction to try to mitigate the risk of lymphedema. In conclusion, while awaiting randomised data to inform us of the necessity of cALND for women with persistently positive nodes following NAC, current guidelines recommend nodal clearance by ALND. However, the survival benefit and regional recurrence rates associated with cALND followed by radiation as opposed to nodal radiation alone in patients with limited residual disease in the axilla following NAC are uncertain, pending the results of the Alliance A11202 trial. Given this clinical equipoise, we propose a nuanced approach to the axilla by utilizing a shared decision model with patients, incorporating targeted SLNB with FS and a completion node dissection when required and desired by the patient, coupled with LVA to mitigate the risk of BCLR in a simple stepwise treatment pathway.

## Figures and Tables

**Table 1:** Patient Demographics.

Demographics	(N, %)
**Age (mean, range)**	49.9 (32–75)
**Menopausal Status**	
**Pre-Menopause**	11 (73.3%)
**Post-Menopause**	4 (26.7%)
**BMI (kg/m^2^)**	
**Non-Obese (BMI < 30)**	11 (73.3%)
**Obese (BMI > 30)**	4 (26.7%)
**Tumour Characteristics**	
**Histological Subtype**	
**Ductal**	12 (80.0%)
**Lobular**	1 (6.7%)
**Not Specified**	2 (13.3%)
**Receptor Subtype**	
**ER/PR Positive HER-2 Negative**	4 (26.7%)
**HER-2 Positive**	5 (33.3%)
**Triple Negative**	6 (40.0%)
**TNM Staging**	
**Tumour (T) Stage**	
**T0**	1 (6.7%)
**T1**	0 (0%)
**T2**	10 (66.7%)
**T3**	4 (20.0%)
**T4**	1 (6.7%)
**Nodal (N) Stage**	
**N1**	11 (73.3%)
**N2**	4 (26.7%)
**N3**	0 (0%)
**Clinical Stage**	
**I**	0 (0%)
**II**	8 (53.3%)
**III**	7 (46.7%)
**IV**	0 (0%)

**Table 2:** Patient-Reported Outcomes with LYMQOL-ARM ^1^.

Metric	All Patients N = 15	cALND and LVA N = 11	SLNB Only N = 4	Patients without Lymphedema N = 14	Patient with Lymphedema N = 1
**Function**	1.1 ± 0.2	1.2 ± 0.2	1.1 ± 0.1	1.1 ± 0.2	1.5
**Appearance**	1.1 ± 0.3	1.2 ± 0.3	1.0 ± 0.0	1.1 ± 0.3	1
**Symptoms**	1.4 ± 0.4	1.4 ± 0.4	1.5 ± 0.5	1.4 ± 0.3	2.3
**Mood**	1.2 ± 0.4	1.1 ± 0.2	1.1 ± 0.2	1.2 ± 0.4	1.2
**Overall Quality of Life**	8.1 ± 0.9	8.1 ± 0.6	8.1 ± 0.6	8.1 ± 0.9	8

^1^ Function, appearance, symptoms, and mood were rated on a score from 1–4 with lower scores indicating less severe symptoms: (1)—not at all, (2)—a little, (3)—quite a bit, and (4)—a lot. Quality of life was measured on a scale from 0–10, where 0 was poor and 10 is excellent.

## Data Availability

The data presented in this study are available on request from the corresponding author.

## Supplementary Materials

The following supporting information can be downloaded at: https://www.mdpi.com/article/10.3390/curroncol30040306/s1, Table S1: Overview of Axillary Surgery and Final Pathology of Regional Nodes.
