# Supplementary material for: Incorporating Lymphovenous Anastomosis in Clinically Node-Positive Women Receiving Neoadjuvant Chemotherapy: A Shared Decision-Making Model and Nuanced Approached to the Axilla
Source: Curr Oncol. 2023 Apr 3;30(4):4041–51. doi: 10.3390/curroncol30040306 (PMC10137272; doi:10.3390/curroncol30040306)
Supplement: Supplementary file 1 [file curroncol-30-00306-s001.zip › curroncol-2210984-supplementary.pdf]

Supplementary Data

Table S1: Overview of Axillary Surgery and Final Pathology of Regional Nodes

Patient	Axillary Surgery	Number of SLN Harvested	Number of Positive SLN Harvested	Total Number of Lymph Nodes Harvested (SLNB + ALND)	Total Number of Positive Lymph Nodes (SLNB + ALNB)	Macroscopic	Microscopic	Isolated Tumour Cells	TMN Staging (8th Edition)
1	ALND + LVA	-	-	18	0	0	0	0	ypT0N0
2	ALND + LVA	-	-	15	0	0	0	0	ypT0N0
3	ALND + LVA	-	-	28	3	0	1	2	ypT1N1mi
4	ALND + LVA	-	-	17	0	0	0	0	ypT2N0
5	ALND + LVA	-	-	12	0	0	0	0	ypT2N0
6	ALND + LVA	-	-	16	1	1	0	0	ypTxN1
7	ALND + LVA	-	-	19	2	0	0	2	ypT1N0(i+)
8	SLNB with FS --> ALND + LVA	3	2	12	9	9	0	0	ypT2N2
9	SLNB with FS --> ALND + LVA	3	2	27	26	26	0	0	ypT3N3
10	SLNB with FS --> ALND+ LVA	4	3	16	4	4	0	0	ypT2N2
11	ALND + LVA	-	-	10	0	0	0	0	ypT1N0
12	SLNB with FS	4	0	4	0	0	0	0	ypT0N0
13	SLNB with FS	5	0	5	0	0	0	0	ypT1N0
14	SLNB with FS	4	3	4	3	1	0	2	ypT1N1
15	SLNB with FS	4	0	4	4	0	0	0	ypT1N0
